# Proliferation and apoptosis after whole-body irradiation: longitudinal PET study in a mouse model

**DOI:** 10.1007/s00259-023-06430-x

**Published:** 2023-10-05

**Authors:** Maria Meindl, Alexandra Bläske, Katja Steiger, Simon Lindner, Felix Lindheimer, Kirsten Lauber, Nikko Brix, Barbara von Ungern-Sternberg, Rosel Oos, Giovanna Palumbo, Guido Böning, Simone Schüle, Matthäus Majewski, Matthias Port, Sibylle Ziegler, Peter Bartenstein

**Affiliations:** 1 Department of Nuclear Medicine, LMU University Hospital, LMU Munich, Munich, Germany; 2 Comparative Experimental Pathology, Institute of Pathology, TU Munich, Munich, Germany; 3 Department of Radiotherapy and Radiation Oncology, LMU University Hospital, LMU Munich, Munich, Germany; 4 Department of Radiology, Bundeswehr Hospital, Ulm, Germany; 5 Department of Urology, Bundeswehr Hospital, Ulm, Germany; 6 Bundeswehr Institute of Radiobiology, Munich, Germany

**Keywords:** Apoptosis, Proliferation, PET, Irradiation

## Abstract

**Purpose:**

A reliable method for regional in vivo imaging of radiation-induced cellular damage would be of great importance for the detection of therapy-induced injury to healthy tissue and the choice of adequate treatment of radiation emergency patients in both civilian and military events. This study aimed to investigate in a mouse model if positron emission tomography (PET) imaging with proliferation and apoptosis markers is potentially suitable for this purpose.

**Methods:**

Four groups, including twenty mice (wild-type C57BL/6) each, were whole-body irradiated with 0 Gy, 0.5 Gy, 1 Gy, and 3 Gy and examined by PET over a six-month period at defined time points. 3'-[^18^F]fluoro-3'-deoxythymidine ([^18^F]FLT) and 2-(5-[^18^F]fluoropentyl)-2-methyl malonic acid ([^18^F]ML-10) were used to visualise proliferation and apoptosis. Regional standard uptake values were compared with respect to irradiation dose over time. Histologic data and peripheral blood cell values were correlated with the PET results.

**Results:**

The hematopoietic bone marrow showed a significantly increased [^18^F]FLT signal at early time points after radiation exposure (day 3 and day 7). This correlated with blood parameters, especially leukocytes, and histological data. A significantly increased [^18^F]FLT signal also occurred in the gastrointestinal tract and thymus at early time points. An increased [^18^F]ML-10 signal related to irradiation doses was observed in the bone marrow on day 8, but there was a high variability of standard uptake values and no correlation with histological data.

**Conclusion:**

[^18^F]FLT showed potential to visualise the extent, regional distribution and recovery from radiation-induced cellular damage in the bone marrow, gastrointestinal tract and thymus. The potential of [^18^F]FLT imaging to assess the extent of bone marrow affected by irradiation might be especially useful to predict the subsequent severity of hematopoietic impairment and to adapt the therapy of the bone marrow reserve. [^18^F]ML-10 PET proved to be not sensitive enough for the reliable detection of radiation induced apoptosis.

**Supplementary Information:**

The online version contains supplementary material available at 10.1007/s00259-023-06430-x.

## Introduction

In vivo imaging of radiation-induced cellular damage is a challenging topic on which substantial work has been performed over the last decades, but recent progress has been limited [1–3]. It could help in the detection of therapy-induced injury to healthy tissue, such as bone marrow toxicity during radionuclide treatment. In addition, it could be useful for the management of radiation accidents, which can occur in the context of both, civilian and military events and may result in radiation syndrome and the need for radioprotective drugs [4, 5].

Acute radiation syndrome consists of subsyndromes, namely hematopoietic, gastrointestinal, cutaneous and cardiovascular syndrome [6–8]. Chronic or delayed effects of the acute radiation syndrome are defective lymphocyte reconstitution and long-term T-cell deficiencies with subsequent immune insufficiency and dampen protective immune responses. This can lead to increased incidence of infections, inflammation and cancer [7]. A better understanding and even visualisation could help to guide patients and to prevent medications to treat this disease [9].

Exposure to ionising radiation activates numerous cascades and processes in the organism, including cell proliferation and apoptosis [10]. These two cellular mechanisms describe responses that avoid and compensate lethal organ damage and can be an indicator for the extent and recovery of cellular damage [11]. According to previous findings, increased cell proliferation and apoptosis occur in the context of exposure to radioactive irradiation especially in the bone marrow and gastrointestinal tract [12–15].

In this work, we evaluate the potential of the in vivo imaging method PET to visualise radiation-induced cell proliferation and apoptosis in a mouse model. Therefore, C57BL/6 mice received whole-body irradiation with different doses followed by PET scans at defined time points over six months using the proliferation tracer 3'-[^18^F]fluoro-3'-deoxythymidine ([^18^F]FLT) and the apoptosis tracer 2-(5-[^18^F]fluoropentyl)-2-methyl malonic acid ([^18^F]ML-10). Both tracers have previously been used in preclinical and clinical studies mostly in the context of assessing cancer treatment response [16–23]. The results predominantly indicated that the tracers are suitable for imaging increased radiation-induced cell proliferation and apoptosis and led to the conclusion that they might be also appropriate for our research question. Moreover, in a previous preclinical study, [^18^F]FLT was already successfully used to visualise cell proliferation in a mouse model after whole-body irradiation of up to 8 Gy [10]. Reference examination methods such as histology, immunohistochemistry and blood analysis were used to investigate whether PET with the tracer [^18^F]FLT and [^18^F]ML-10 has the potential to distinguish regionally increased apoptosis and proliferation processes in irradiated mice (0.5 Gy, 1 Gy, 3 Gy) from physiological proliferation and apoptosis processes in sham-irradiated mice (0 Gy).

## Material and methods

### Radiochemistry

Radiosynthesis of [^18^F]FLT and [^18^F]ML-10 was performed with established standardised protocols presented in the supplement. The procedures yielded a radiochemical purity exceeding > 99%.

### Animals

9 to 12 weeks old male C57BL/6 wild-type mice (Charles River Laboratories, Sulzbach, Germany) were investigated. They were housed in a temperature- and humidity-controlled environment with a 12-h light-dark cycle and had free access to food (Ssniff) and water. All experiments were performed in compliance with the National Guidelines for Animal Protection, Germany, with the approval of the regional animal committee (Regierung Oberbayern, ROB-55.2-2532.Vet_02-17-229) and were overseen by a veterinarian.

### Study design

The study design consisted of a PET study and a correlation study that included examinations regarding histology and immunohistochemistry as well as blood parameters.

**Fig. 1:**
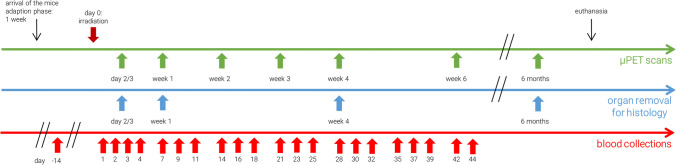
Overview of the timing of the PET study and correlation study

The PET study entailed a longitudinal series of [^18^F]FLT and [^18^F]ML-10 PET sessions performed in sham-irradiated mice (0 Gy) and mice whole-body irradiated with different doses (0.5 Gy, 1 Gy, 3 Gy) (group 1–8), followed by histology and immunohistochemistry for the correlation study. Additional mice served for histological and immunohistochemical examinations at earlier time points (group 9–15). The blood investigations of the correlation study were performed in a longitudinal design with a separate cohort of sham-irradiated and irradiated mice (group 16–18). A detailed overview of the different groups of mice and time points is provided in Table 1 and Fig. 1.

**Table 1:**
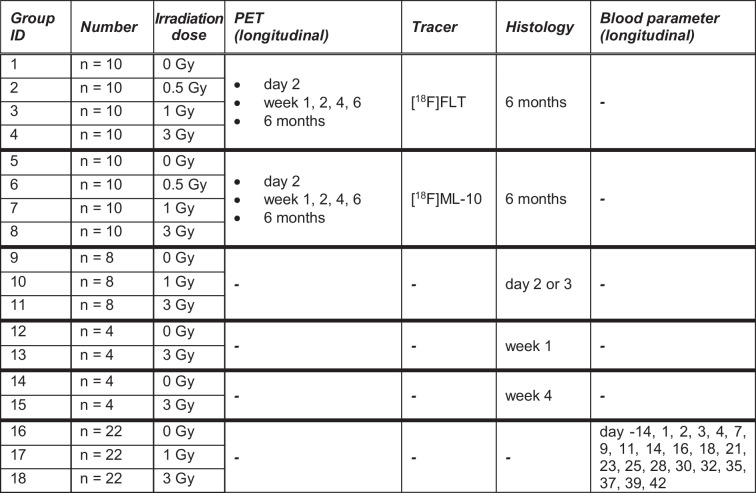
Overview of mouse numbers, irradiation doses and timing of the PET study and correlation study. The time points regarding PET, histology and blood parameters refer to all mice of the groups that are assigned to the corresponding table cell

### Irradiation

The mice were whole-body irradiated with doses of 0.5 Gy, 1 Gy or 3 Gy using the X-ray research irradiator cabinet RS225 or the irradiation platform SARRP (X-Strahl, Ratingen, Germany). The sham-irradiated mice were transported to the irradiation systems in the same way as the irradiated mice and placed next to the irradiation field to ensure that they were exposed to a similar stress level.

### PET imaging

#### PET data acquisition and reconstruction

PET imaging was performed simultaneously in four mice using a Siemens Inveon Dedicated PET (Siemens, Erlangen, Germany) for the [^18^F]FLT scans and a Mediso nanoScan PET/CT (Mediso, Münster, Germany) for the [^18^F]ML-10 scans. A fifteen-minute transmission scan with an external ^57^Co point source (Siemens Inveon Dedicated PET) or a low-dose CT (Mediso nanoScan PET/CT) served for attenuation correction. The PET data were acquired in 3-dimensional list-mode after 14.5 ± 1.1 MBq of the tracer had been injected into a tail vein. The [^18^F]FLT PET was performed 60 to 90 min p.i., the [^18^F]ML-10 PET was performed 120 to 150 min p.i. The acquired data were reconstructed with attenuation and scatter correction using the software (Inveon Acquisition Workplace 1.528 Service Pack 1, Nucline NanoScan 3.04.018.0000) and reconstruction algorithms provided by the vendors: ordered subset expectation maximisation algorithm with 4 iterations and 26 subsets (32 MAP iter, pixel size (x, y, z): 0.8, 0.8, 0.8 [mm], matrix size (x, y, z): 128, 128, 159 [mm]) on the Siemens Inveon Dedicated PET and 4 iterations and 6 subsets (pixel size (x, y, z): 0.4, 0.4, 0.4 [mm], matrix size (x, y, z): 212, 212, 239 [mm]) on the Mediso nanoScan PET/CT. Reconstruction parameters for the two devices were chosen for equivalent spatial resolution.

#### PET image analysis

Images were analysed using the freeware AMIDE. Regions of increased [^18^F]FLT and [^18^F]ML-10 uptake were visually assessed. Subsequently, spheres or manually defined geometric shapes were placed as volumes of interest (VOIs) in these regions and standard uptake values (SUVs) were calculated with the maximum voxel values of the VOIs. They were normalised with the SUVs determined in the neck area of the mice, since no specific tracer binding was expected in the fat and muscle tissue of the neck.

### Histology and immunohistochemistry

Histology and immunohistochemistry were performed by a board certified veterinary pathologist (KS). The mice were killed by cervical dislocation while deeply anesthetised. The liver, kidneys, spleen, lungs, heart, gastrointestinal tract, stomach, testis, salivary glands, thymus and bone (femur, vertebrae) were dissected, paraffin embedded, and sectioned. Hematoxylin eosin staining was done as well as Ki-67 staining to visualise proliferation and cleaved Caspase3 staining to visualise apoptosis. For the analyses, the stained sections were digitalized and magnified 20 times at maximum resolution (0.252 µm per pixel). Then, five randomly selected field of views were observed. In addition to histological descriptive evaluation, proliferative and apoptotic processes in each section were assessed semiquantitatively. For proliferation, scores were assigned from 0 to 10. 5 corresponded to the expected number of mitoses in the observed organ, 0 to 4 described graded decreases in mitoses, whereas 6 to 10 described graded increases in mitoses. For apoptosis, values from 0 to 3 were assigned. 0 indicated no apoptosis, 3 indicated numerous apoptosis.

### Blood sampling

Blood samples were collected by puncturing the facial vein dorsocaudal to the hair vertebra on the mandible as the mice were under isoflurane anaesthesia. K3 EDTA was used as anticoagulant. The examination of the blood samples regarding the number of leukocytes, erythrocytes and thrombocytes was performed with the mouse blood analyser scil VET abc (scil animal care company GmbH, Viernheim, Germany). For manual leukocyte differentiation, blood smears were stained with a Wright-Giesma staining solution. More details regarding the blood sampling process are presented in the supplement.

### Statistics

GraphPad Prism version 9.1.2 (226) (GraphPad Software, San Diego, United States) was used for statistical testing. Group comparisons of VOI-based PET results regarding irradiation doses and measurement times were assessed by 2-way ANOVA. The semiquantitative evaluations of histology and immunohistochemistry were compared using 1-way ANOVA. For correlation analyses, Pearson correlation coefficients r were calculated. P values less than 0.05 were considered significant (*p* < 0.05: *, *p* < 0.01: **, *p* < 0.001: ***, *p* < 0.0001: ****).

## Results

### [^18^F]FLT PET

**Fig. 2:**
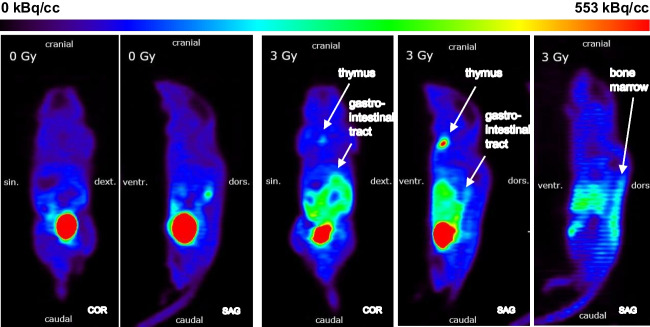
[^18^F]FLT uptake in thymus, gastrointestinal tract and bone marrow at day 3 after irradiation. Left: Coronal and sagittal slices of a sham-irradiated (0 Gy) mouse (group 1). Right: One coronal and two sagittal slices of a with 3 Gy irradiated mouse (group 4)

**Fig. 3:**
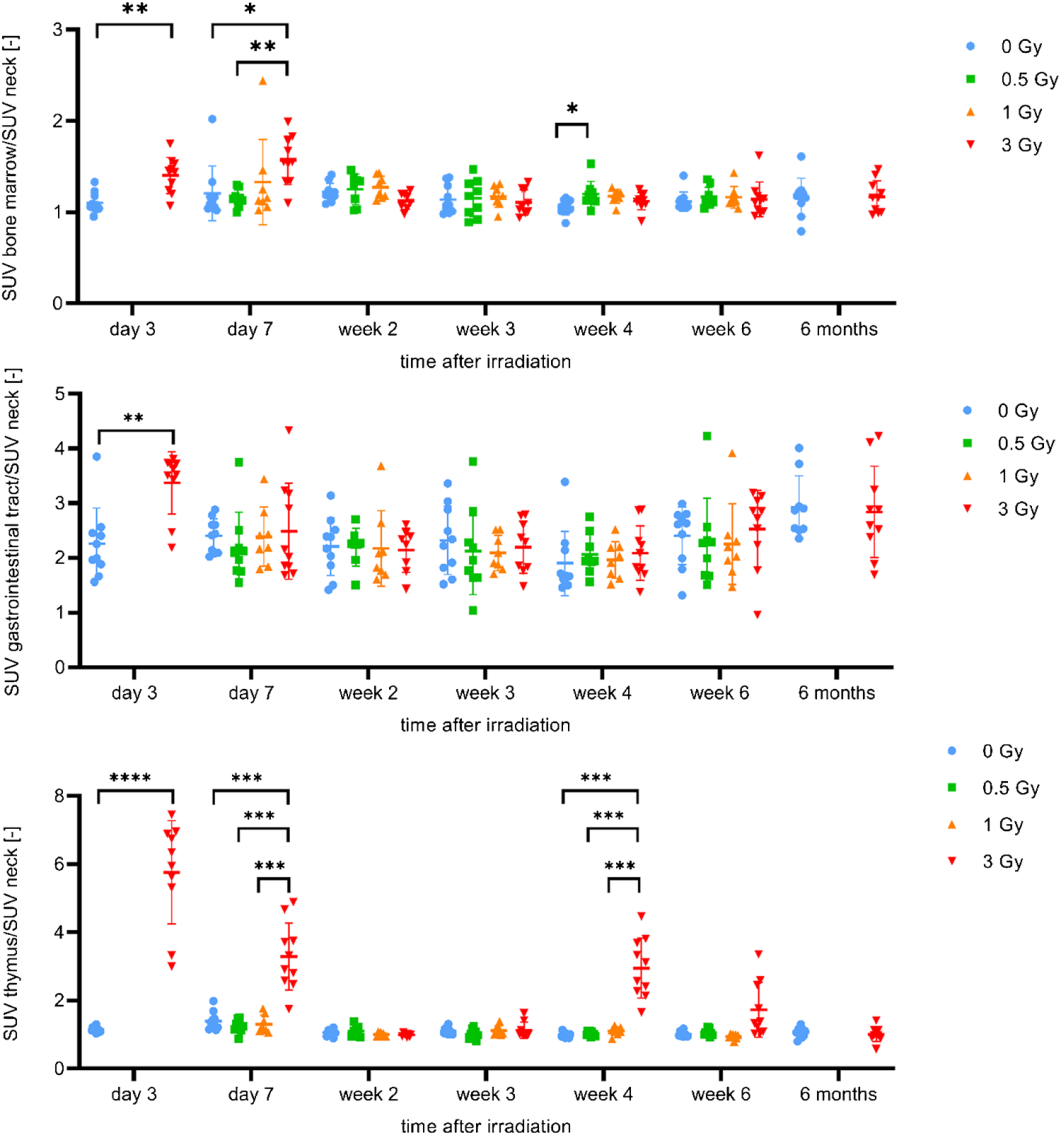
Regional proliferation signal up to 6 months after irradiation. Longitudinal VOI-based group comparisons (group 1–4) of normalised [^18^F]FLT SUVs in bone marrow, gastrointestinal tract and thymus (2-way ANOVA)

[^18^F]FLT PET images of the irradiated mice showed an increased signal in the thymus, gastrointestinal tract and bone marrow at early time points (day 3 and 7 after irradiation) (Fig. 2). Data of the mice irradiated with 0.5 Gy and 1 Gy (groups 2 and 3) are not available on day 3 (synthesis failure) and 6 months (premature death) after irradiation. The other groups had up to seven successful scans per mouse. The SUVs of the mice irradiated with 3 Gy were significantly increased compared with those of the sham-irradiated mice (Fig. 3). 4 weeks after irradiation, an increased [^18^F]FLT uptake occurred again in the thymus of the mice irradiated with 3 Gy, SUVs were significantly increased compared to the other groups (Fig. 3).

### [^18^F]ML-10 PET

**Fig. 4:**
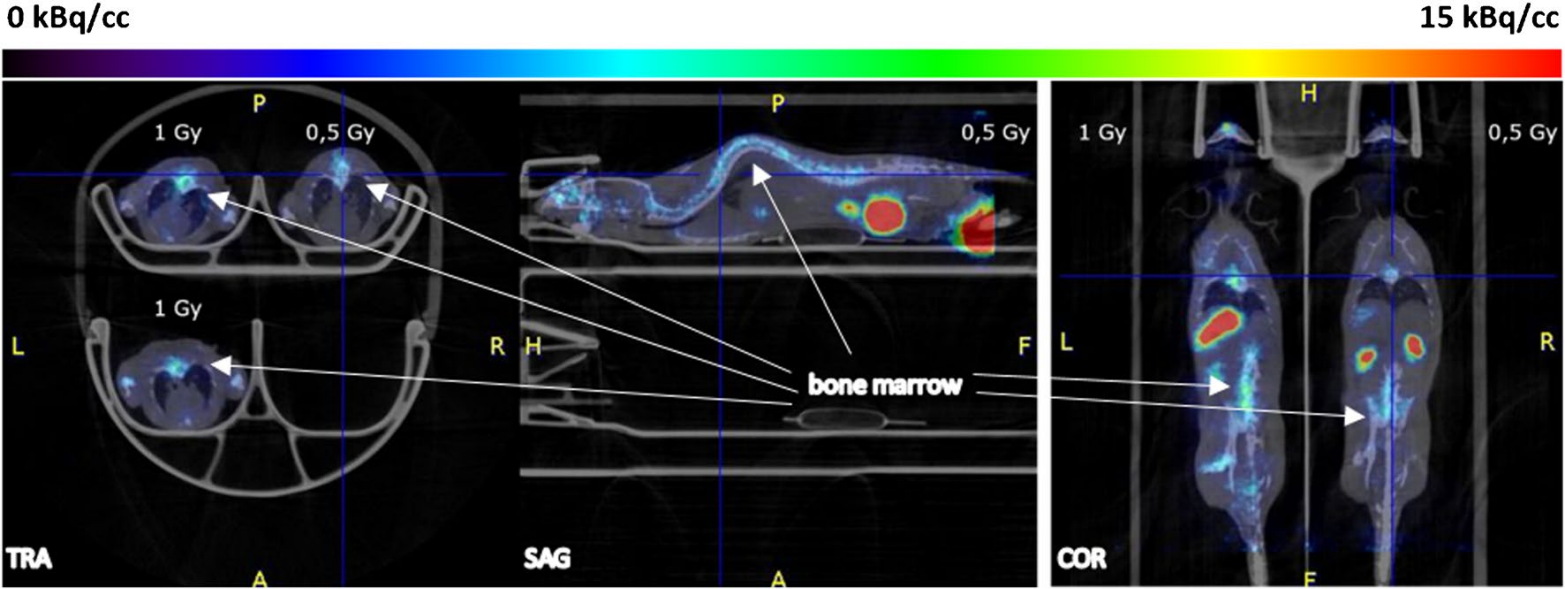
[^18^F]ML-10 uptake in bone marrow at day 8 after irradiation in with 0.5 and 1 Gy irradiated mice (group 6 and 7)

**Fig. 5:**
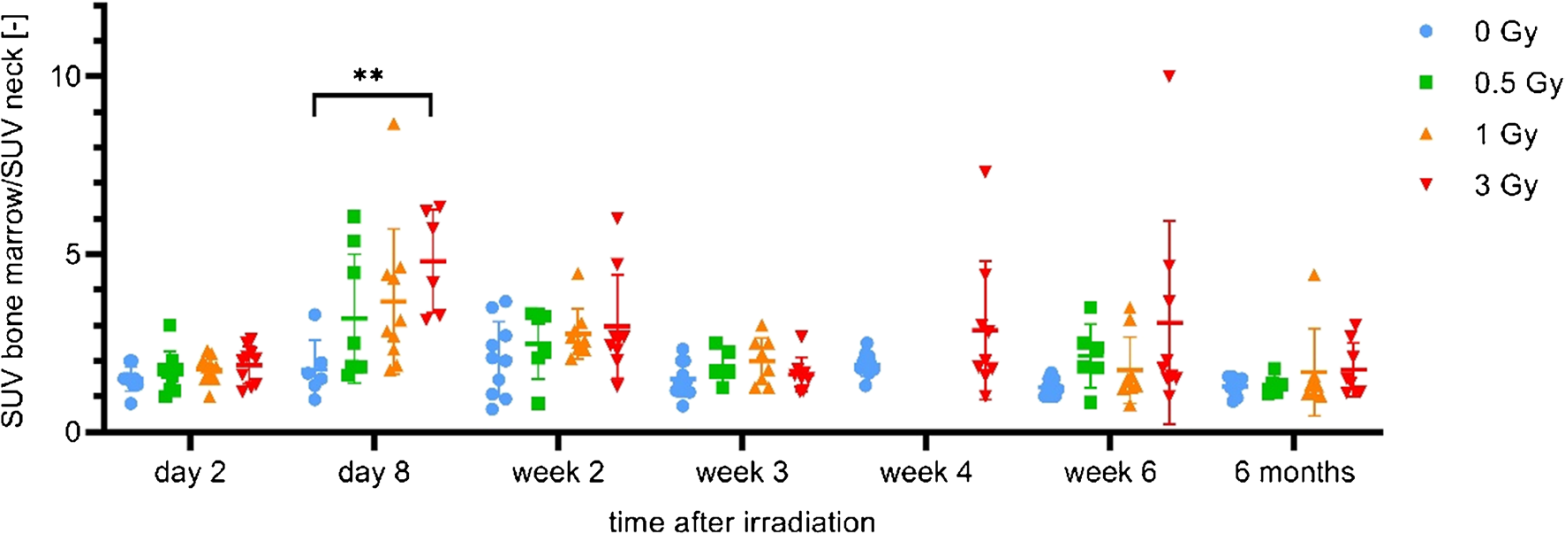
Apoptosis signal up to 6 months after irradiation. Longitudinal VOI-based group comparisons (group 5–8) of normalised [^18^F]ML-10 SUVs in bone marrow (2-way ANOVA)

There were up to six successful scans per mouse. Data of the mice irradiated with 0.5 Gy and 1 Gy (groups 6 and 7) are not available at week 4 after irradiation because of synthesis failure. [^18^F]ML-10 PET images of the irradiated mice demonstrated an increased, dose dependent signal in the bone marrow on day 8 after irradiation (Fig. 4). SUVs confirmed this visual impression, however, they showed a high variability (Fig. 5). A significant difference was observed between the SUVs of the mice irradiated with 3 Gy and those of the sham-irradiated mice on day 8.

### Histology and immunohistochemistry

#### Bone marrow

**Fig. 6:**
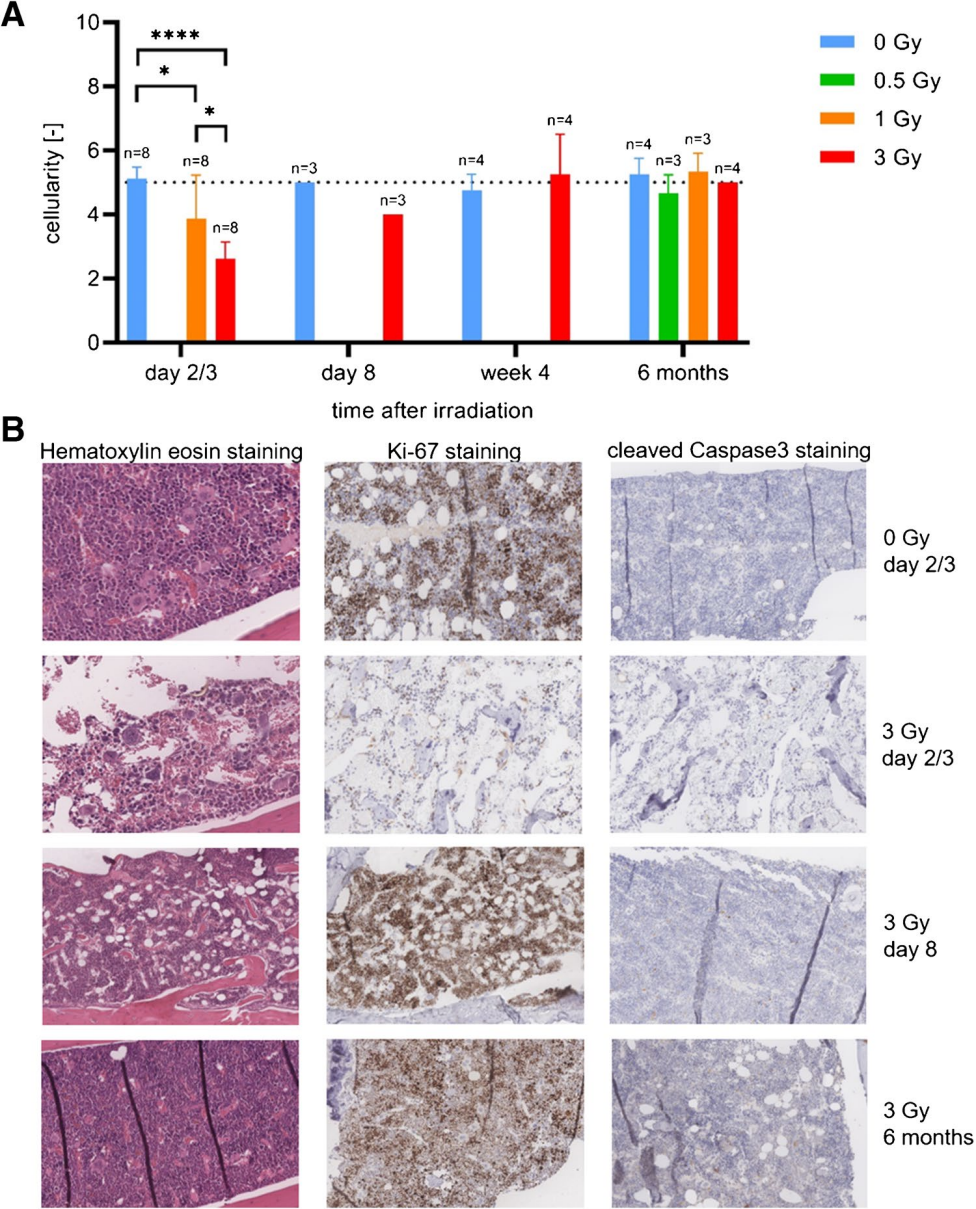
**A** Longitudinal group comparisons (group 1–15) of cellularity in bone marrow (1-way ANOVA). Lower mouse numbers in group 12 and 13 are due to spontaneous death. **B** Haematopoiesis and proliferation in bone marrow at day 2/3, day 8 and 6 months after irradiation (hematoxylin eosin staining and Ki-67 staining)

The visual analysis and semiquantitative evaluation of histology and immunohistochemistry of the bone marrow demonstrated a loss of haematopoiesis on day 2 and 3 after irradiation, associated with a decreased proliferation activity and a strong decrease in cell number (Fig. 6). Regarding apoptotic cell deaths, no differences were observed in either 1 Gy or 3 Gy irradiated mice compared to sham-irradiated mice. On day 8 after irradiation, slightly more fatty marrow was visible in the mice irradiated with 3 Gy than in the sham-irradiated mice. In addition, a strong proliferation activity was observed in the irradiated mice. With regard to apoptotic processes, there were no differences to the sham-irradiated mice. In week 4 after irradiation, haematopoiesis had mostly recovered, 6 months after irradiation no differences were observed between the sham-irradiated and irradiated mice (Fig. 6B).

#### Gastrointestinal tract

**Fig. 7:**
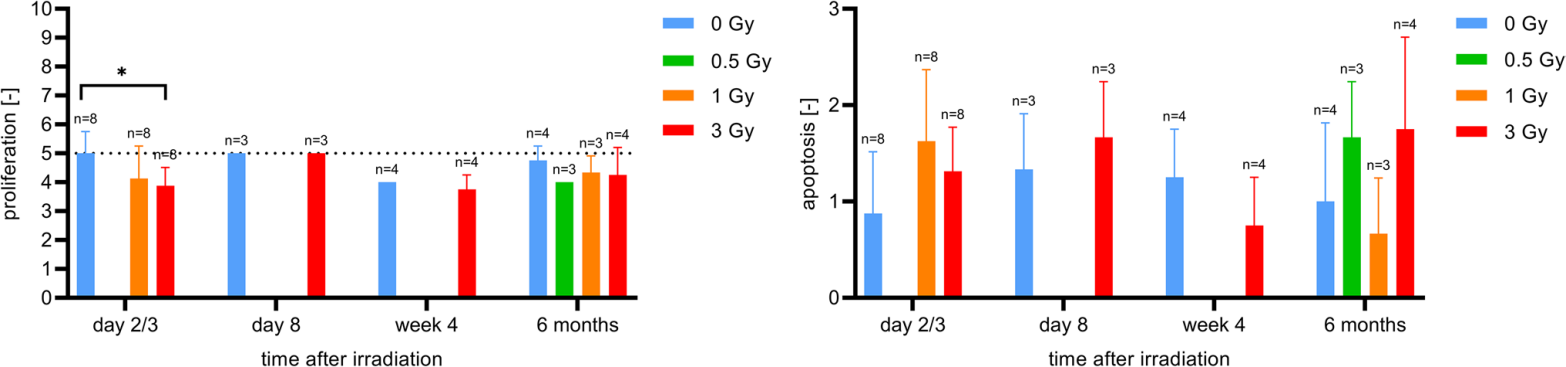
Longitudinal group comparisons (group 1–15) of proliferation and apoptosis in gastrointestinal tract (1-way ANOVA). Lower mouse numbers in group 12 and 13 are due to spontaneous death

Regarding the gastrointestinal tract, histology and immunohistochemistry showed strong individual differences across all irradiation doses and time points with respect to both proliferative and apoptotic processes (Fig. 9 in supplement). There was a trend towards slightly more apoptosis and less proliferation after irradiation, but the differences were small (Fig. 7).

#### Thymus

Histological and immunohistochemical examinations of the thymus showed an extensive destruction of the cortex and a loss of corticomedullary distinction (decreased numbers of cortical and medullary lymphocytes, little or no distinction between cortical and medullary cellularity/cell density [24]) on day 2 after irradiation, especially in the mice irradiated with 3 Gy (Fig. 10 in supplement). On day 3, this effect was already regressing, 4 weeks after irradiation, the cortex and the corticomedullary ratio had fully recovered. With respect to apoptotic cell death, no differences were observed between the irradiated and sham-irradiated mice.

### Blood sampling

**Fig. 8:**
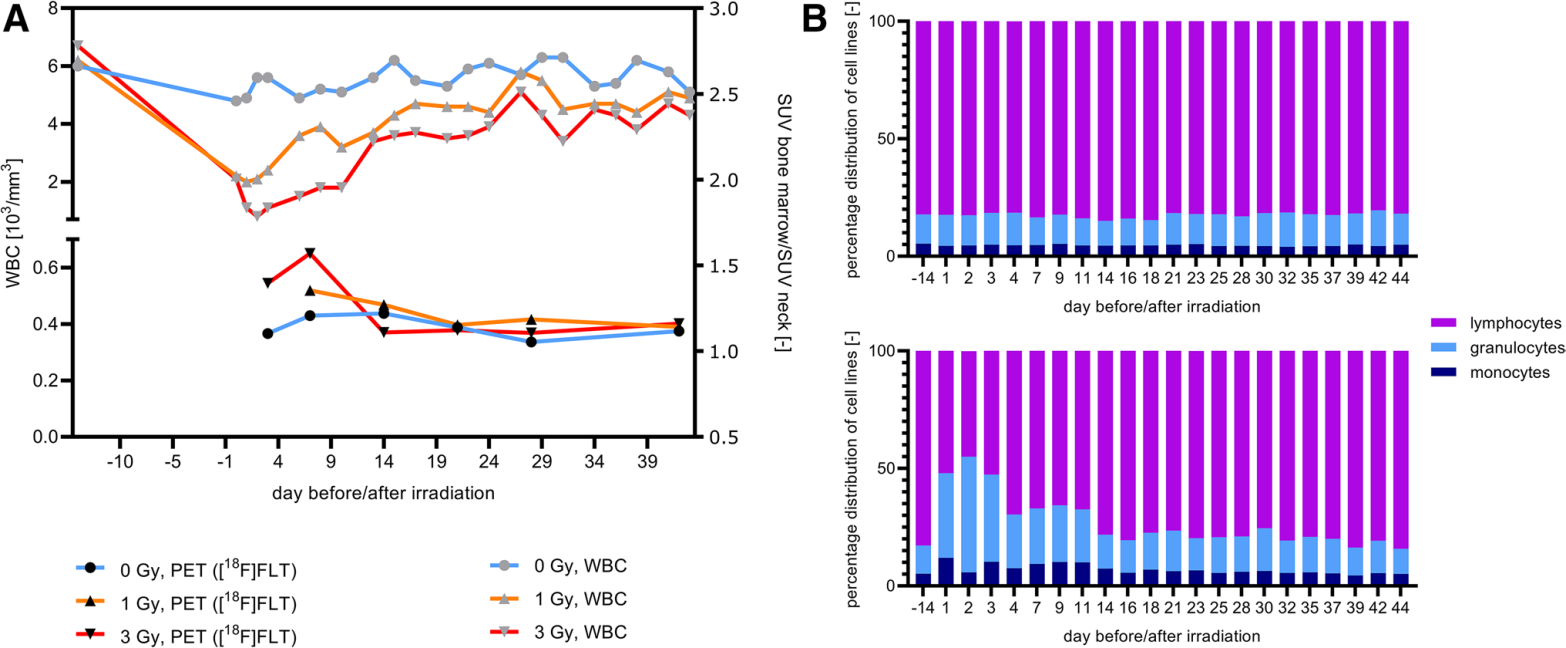
**A** Longitudinal group comparisons (group 16–18) of average numbers of leukocytes (WBC) (left axis) correlated with longitudinal VOI-based group comparisons (group 1–4) of normalised and averaged [^18^F]FLT SUVs in bone marrow (right axis). **B** Longitudinal comparison of percentage distribution of cell lines in sham-irradiated mice (upper row) and with 3 Gy irradiated mice (lower row)

Analysis of blood parameters showed a decrease in the average numbers of erythrocytes, leukocytes and thrombocytes correlating with the irradiation dose in the first days after irradiation (Fig. 8A and Fig. 11 in supplement). Depending on the blood cell type, the values returned to those of the sham-irradiated mice after 2 to 4 weeks. Examinations of the cell lines of leukocytes showed that the percentage distribution of the lymphocytes, granulocytes and monocytes changed in the irradiated mice. While the leukocytes in the sham-irradiated mice were composed of approximately 80% lymphocytes, 15% granulocytes and 5% monocytes, this distribution shifted in the irradiated mice and there was a percentage increase in granulocytes and a percentage decrease in monocytes within the first days after irradiation (Fig. 8B). The changes in absolute numbers of cell lines are presented in the supplement (Fig. 12 in supplement).

## Discussion

PET data as well as histological findings and blood parameters indicate that the whole-body irradiation of the mice caused cellular damage and initiated subsequent proliferative processes.

PET images showed significantly increased [^18^F]FLT uptakes in the bone marrow of the mice irradiated with 3 Gy on day 2 and 3 after irradiation, but no increased [^18^F]ML-10 uptakes. In contrast, one week after irradiation, both signals increased significantly. Histological and immunohistochemical findings of the bone marrow showed a loss of haematopoiesis on day 2 and 3 after irradiation, associated with a decreased proliferation activity and a strong decrease in cell number. In contrast, on day 8 after irradiation an increased proliferation activity was visible and haematopoiesis recovered. This correlated with the blood parameters, especially the leukocytes. Depending on the irradiation dose, they showed a strong decrease in their average number with a nadir on day 3 after irradiation. After that, the average leukocyte numbers increased, around day 28 after irradiation they became equal to those of the sham-irradiated mice. At this time, histological evaluations also showed that haematopoiesis had largely recovered.

The histological and immunohistochemical analyses suggest that apoptosis played only a minor role at the time points examined [24]. It is hypothesised that necrosis rather than apoptosis was present as cell death mechanism at day 2 and 3 after irradiation, leading to strong cell number reductions in both blood parameters and histological data. That only few apoptotic processes occurred was also confirmed by the [^18^F]ML-10 PET images, in that no increased uptakes were visible on day 2 after irradiation. Also, it could be possible that apoptosis occurred at earlier time points (e.g. in the first 24 h after irradiation) and that the resulting dead cells were already cleared by the time of the first [^18^F]ML-10 PET scan. From day 3 after irradiation, the cells that were not destroyed started to proliferate, which was confirmed by the [^18^F]FLT PET images as well as by the blood parameters and histological findings. The increased [^18^F]ML-10 uptake on day 8 after irradiation was unexpected and needs further investigation.

Regarding the gastrointestinal tract, discrepancies between the PET data and histological data have occurred. While increased [^18^F]FLT uptakes were visible on the PET images of irradiated mice at early time points after irradiation, the histological and immunohistochemical examinations did not show any relevant differences with respect to proliferative processes between the irradiated and sham-irradiated mice. This could be due to the fact that only small sections of the intestine were examined in histology, whereas the entire intestine was visualised on the PET images. Overall, it can be assumed that proliferation occurred in the gastrointestinal tract due to irradiation, as this is also confirmed by literature [10, 12]. However, it should also be considered that proliferation in cells of the spleen and mesenteric lymph nodes could have been the reason for the increased [^18^F]FLT signal. This needs to be investigated further.

In the thymus, significantly increased [^18^F]FLT uptakes occurred in the mice irradiated with 3 Gy on day 3 and 7 after irradiation. This was consistent with the results of the histological and immunohistochemical examinations of the thymus: The extensive destruction of the cortex and the loss of corticomedullary distinction in mice irradiated with 3 Gy completely regressed after 4 weeks. This indicated strong proliferation, which was shown in PET by significantly increased [^18^F]FLT SUVs.

Regarding military medical indications and nuclear disasters, PET with the tracer [^18^F]FLT could be useful to detect the extent of damage to the hematopoietic bone marrow after partial or whole-body exposure to irradiation. The necessary treatment could be then individually adapted to the still intact haematopoiesis. For this purpose, a PET scan should be performed on day 3 after irradiation exposure. Clinically preferable might be an even earlier PET scan to guide therapy right after an exposure. However, [^18^F]FLT PET scans before day 3 after irradiation were not part of the study.

Considering the correlation of the [^18^F]FLT PET signal in the hematopoietic bone marrow and the blood parameters over time, it can be concluded that PET with the tracer [^18^F]FLT has the potential to be used as a biomarker for bone marrow recovery. Therefore, there should be performed another PET scan two weeks after irradiation exposure, in addition to the regular examination of blood samples. This could allow to visualise the regional and global recovery of the bone marrow.

PET with the tracer [^18^F]FLT also showed potential to simultaneously detect abdominal irradiation damage, although correlation with histological data should be investigated more specifically.

## Conclusion

Overall, it can be concluded that PET with the tracer [^18^F]FLT showed potential to visualise and detect proliferative processes due to irradiation induced cell damage. Especially with regard to proliferation in the bone marrow, there was good agreement with the histological findings as well as the blood parameters. In contrast, PET with the tracer [^18^F]ML-10 was not sensitive enough for imaging apoptotic processes due to irradiation induced cell damage. In order to finally assess the potential of this tracer, the extent and time points of apoptosis after whole-body irradiation should be further investigated.

### Supplementary Information

Below is the link to the electronic supplementary material.

Supplementary file1 (DOCX 1.32 MB)

## Data Availability

The data that support the findings of this study are available from the corresponding author (MM) upon reasonable request.
