# Supplementary material for: Proliferation and apoptosis after whole-body irradiation: longitudinal PET study in a mouse model
Source: Eur J Nucl Med Mol Imaging. 2023 Oct 5;51(2):395–404. doi: 10.1007/s00259-023-06430-x (PMC10774227; doi:10.1007/s00259-023-06430-x)
Supplement: Supplementary file 1 — Supplementary file1 (DOCX 1.32 MB) [file 259_2023_6430_MOESM1_ESM.docx]

**SUPPLEMENT**

**Materials and Methods**

*Radiosynthesis of [^18^F]FLT.*

Automated production of [^18^F]FLT was performed on a GE FASTlab^TM^ synthesiser with single-use disposable cassettes. The pre-filled vial containing the DMTr-Boc-Nosyl-FLT precursor and a water bag (water for injection) were assembled on the cassette and the cassette was mounted on the synthesiser according to the set-up instructions. The FASTlab^TM^ control software prompts were followed to run the cassette test and to start the synthesis. No carrier added [^18^F]fluoride was produced via ^18^O(p, n)^18^F reaction by proton irradiation of ^18^O-enriched water and delivered to the ^18^F incoming reservoir. The precursor is labelled by nucleophilic substitution of the nosylate group by [^18^F]fluoride. The resulting labeled precursor is deprotected by an acidic hydrolysis with phosphoric acid leading to [^18^F]FLT in an organic/aqueous solution. [^18^F]FLT is subsequently trapped on a reverse phase extraction cartridge for pre-purification. [^18^F]FLT is then purified, eluted and formulated into the final product collection vial with ethanol/water solution. RCY 12 ± 2 % (n = 10) non d. c., synthesis time 55 min, RCP > 99 %.

*Radiosynthesis of [^18^F]ML-10.*

[^18^F]ML-10 was synthesised on a Trasis AllinOne (Ans, Belgium) automated synthesis unit (ASU) consisting of 3 manifolds connected in series with a total of 18 valves. The manifolds were clamped into the correct position at the module. All reagents and materials were assembled on the pre-defined positions of the manifold (position 2: eluent, position 5: SAX cartridge, position 8: precursor solution, position 9: HCl syringe, position 11: aq. phosphoric acid bag, position 12: PBS bag, position 13: SPE cartridge, position 15: EtOH, position 16: water). The software prompts were followed, and manual intervention was necessary only during HPLC purification. No carrier added [^18^F]fluoride was produced via ^18^O(p, n)^18^F reaction by proton irradiation of ^18^O-enriched water and delivered to the activity inlet reservoir. The activity was then trapped on a Waters QMA Plus Light Carb cartridge and eluted into the reactor using the eluent solution (12.5 mg K_222_, 12.5 µL 1 M K_2_CO_3_, 187.5 µL H_2_O and 800 µL MeCN). After azeotropic drying, the precursor solution (4 mg ML10 tosylate in 800 µL MeCN) was transferred to the reactor, and the reaction mixture was heated at 125 °C for 10 minutes. The reaction mixture was quenched with aqueous 3 M HCl (0.5 ml) and heated at 125 °C for further 5 minutes. The mixture was diluted with 3.8 ml water and purified via semi-preparative HPLC (Inertsil ODS-4 C18 column, 250 x 10 mm, 5 µm; isocratic elution with 70 % (v/v) H_2_O + 0.1 % (v/v) H_3_PO_4_ (85 wt. % in water) / 30 % acetonitrile; flow: 5 ml/min). The product peak was collected, diluted with 30 ml H_2_O + 0.1 % (v/v) H_3_PO_4_ (85 wt. % in water) and passed through a Waters tC18 SepPak Plus Short cartridge pre-conditioned with 5 ml EtOH and 7 ml H_2_O + 0.1 % (v/v) H_3_PO_4_ (85 wt. % in water). The cartridge was rinsed with 10 ml H_2_O + 0.1 % (v/v) H_3_PO_4_ (85 wt. % in water) and the radiolabeled product was eluted with 1 ml EtOH and diluted with phosphate buffered saline (11 ml). The formulated product solution was transferred to a dispenser and filtered through a Merck 0.22 µm Cathivex-GV sterile filter. The product was obtained in radiochemical yields of 27.0 ± 4.0 % non d.c. (n = 18) within a synthesis time of 60 min. The RCP was >99 %.

*Blood sampling.*

For blood collection, the facial vein was punctured with a 4-5.5 mm lancet at 3-4 mm dorsocaudal to the hair vertebra on the mandible. The blood was collected using a 200 µl microvette, which was carefully swirled right afterwards to distribute the anticoagulant K3 ETDA homogenously. The sample was then set aside for 15-30 minutes to incubate with the anticoagulant. The punctured region of the facial vein was compressed with a sterile gauze for 10-30 seconds. Before determining the number of leukocytes, erythrocytes and thrombocytes using the blood analyser scil VET abc (scil animal care company GmbH, Viernheim, Germany), the sample was again swirled at room temperature. Two measurements were performed per sample. It was considered that the measurement of thrombocytes may be influenced by the blood collection technique, as blood flow may depend on various factors such as vascular congestion and the exact location of the vein puncture. Slow blood flow may result in aggregate formation in the blood sample so that the thrombocyte numbers measured do not reflect the real thrombocyte numbers in the blood sample. Due to the quite uniform average thrombocyte numbers measured in the different groups, it was assumed that the influence of the collection technique was negligible. For manual leukocyte differentiation, 20 µl was pipetted with a micropipette and three blood smears were prepared per blood sample. For fixation, a thin smear (approximately one cell thickness) was prepared on a sterile microscope slide with the aid of a temporary microscope slide or a cover slip. This was set aside to air dry. A buffer solution (0.1 M, pH 6.5) was then prepared: 9.078 g potassium dihydrogen phosphate (KH_2_PO_4_) and 11.576 g disodium hydrogen phosphate dihydrate (Na_2_HPO_4_ x 2 H_2_0) were each mixed with 1000 ml distilled water. Finally, 68.7 ml of the potassium dihydrogen phosphate water solution was mixed with 31.1 ml of the disodium hydrogen phosphate dihydrate water solution. For staining, 1 ml of Wright-Giemsa staining solution was added onto the blood smear, taking care to cover the entire surface. After 3-4 minutes, 2 ml of the buffer solution was added. After another 6-8 minutes, the smear was washed off until the edges turned slightly pink, then the blood smear was set aside to air dry. Based on the stained blood smears, cell morphology could be assessed and cells could be counted and differentiated.

**Results**

*Histology and Immunohistochemistry.*


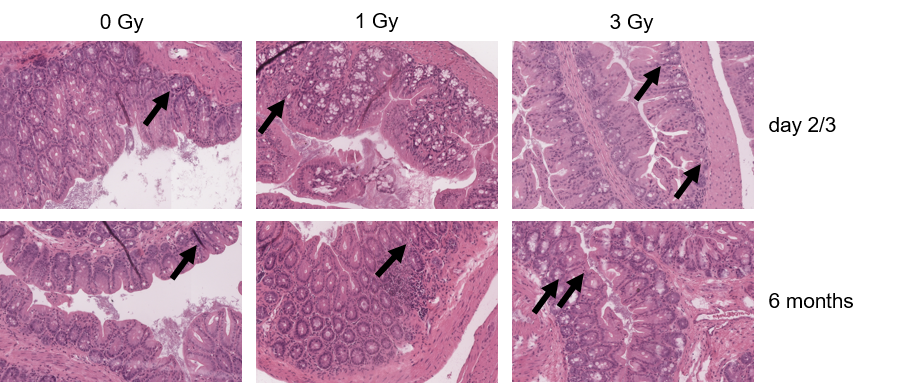


*Figure 9: Proliferation and apoptosis in gastrointestinal tract at day 2/3 and 6 months after irradiation (hematoxylin eosin staining). The arrows symbolize apoptotic bodies which occured across all irradiation doses and time points. There were no clear trends towards more or less apoptosis and prolioferation after irradiation.*

**
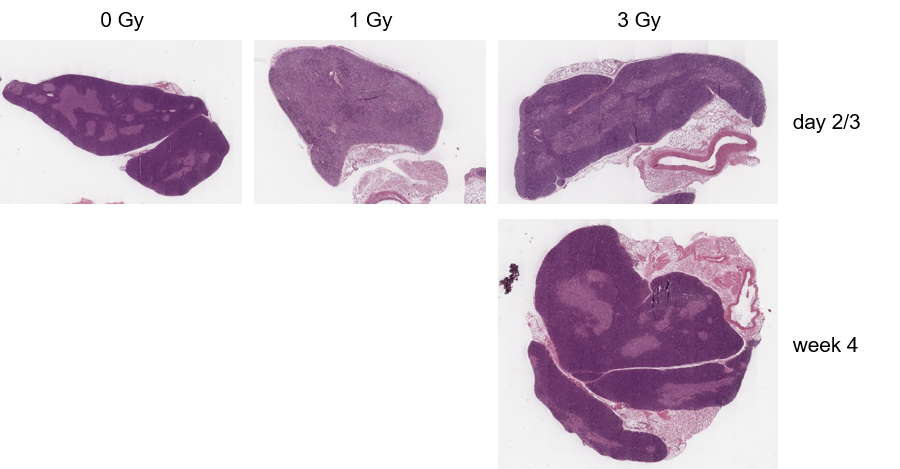
**

*Figure 10: Destruction of the cortex of the thymus. Decreased numbers of cortical and medullary lymphocytes and little or no distinction between cortical and medullary cellularity/cell density (“loss of corticomedullary distinction”) (hematoxylin eosin staining).*

*Blood Sampling.*


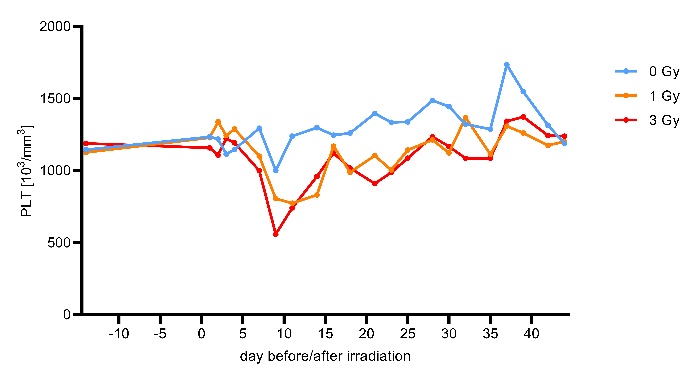

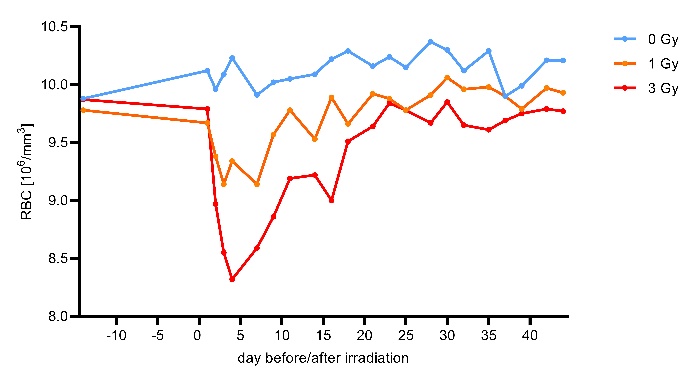


*Figure 11: Longitudinal group comparisons (group 16-18) of average numbers of thrombocytes (PLT) (left) and erythrocytes (RBC) (right). Thrombocytes: strong decrease in the average platelet numbers from day 7 until day 16 after irradiation; influence of the blood collection technique on the platelet numbers was negligible. Erythrocytes: decrease of average numbers within the first days after irradiation, nadir on day 4, values equalize those of the control group (0 Gy) around day 23.*


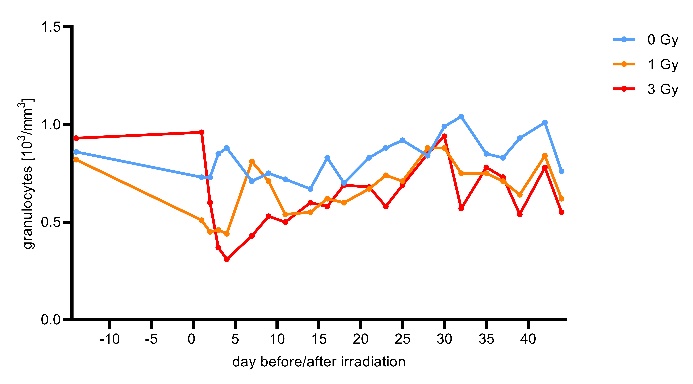

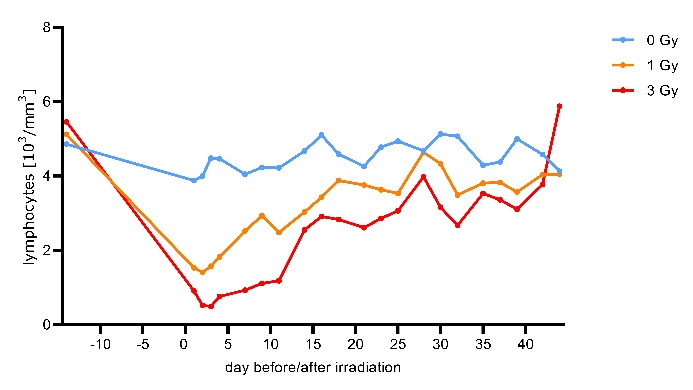


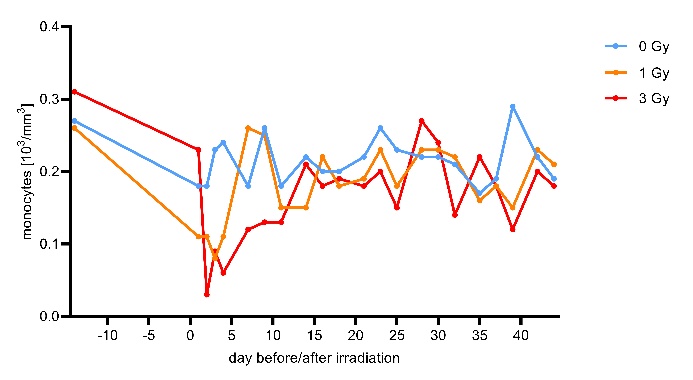


*Figure 12: Longitudinal group comparisons (group 16-18) of average numbers of granulocytes, lymphocytes and monocytes. Granulocytes: strong decrease of average numbers, initial increase for mice irradiated with 3 Gy. Lymphocytes: strong decrease of average numbers immediately after irradiation, values equalized those of the control group (0 Gy) after approximately one month. Monocytes: high variablilty, decrease of average numbers in the first week after irradiation.*
